# Diagnostic characteristics of the 20-minute whole blood clotting test in detecting venom-induced consumptive coagulopathy following carpet viper envenoming

**DOI:** 10.1371/journal.pntd.0011442

**Published:** 2023-06-26

**Authors:** Frank-Leonel Tianyi, Muhammad Hamza, Saidu B. Abubakar, Jaffer Al Solaiss, Anna Trelfa, Hadiza L. Abdullahi, Garba Iliyasu, Nuhu Mohammed, Suleman A. Mohammed, Nicholas R. Casewell, Robert A. Harrison, David G. Lalloo, Ymkje Stienstra, Abdulrazaq G. Habib

**Affiliations:** 1 Centre for Snakebite Research and Interventions, Liverpool School of Tropical Medicine, Liverpool, United Kingdom; 2 Department of Tropical Disease Biology, Liverpool School of Tropical Medicine, Pembroke Place, Liverpool, United Kingdom; 3 Nigeria Snakebite Research and Intervention Centre, Centre for Advanced Medical Research, Bayero University Kano, Nigeria; 4 Infectious Disease and Tropical Medicine Unit, Department of Medicine, College of Health Science, Bayero University Kano, Nigeria; 5 University of Groningen, Department of Internal Medicine/Infectious Diseases, University Medical Centre Groningen, Groningen, The Netherlands; Fundação de Medicina Tropical Doutor Heitor Vieira Dourado, BRAZIL

## Abstract

**Introduction:**

Envenoming by *Echis* spp. (carpet or saw-scaled vipers) causes haemorrhage and coagulopathy and represents a significant proportion of snakebites in the savannah regions of West Africa. Early diagnosis of envenoming is crucial in the management of these patients and there is limited evidence on the utility of the 20-minute whole blood clotting test (20WBCT) in diagnosing venom-induced consumptive coagulopathy (VICC) following envenoming by *Echis ocellatus*.

**Methods:**

A prospective observational cohort study was conducted at the Kaltungo General Hospital in North-eastern Nigeria from September 2019 to September 2021. Standardised 20WBCTs were conducted by trained hospital staff and citrated plasma samples were collected at numerous timepoints. Prothrombin time (PT) and international normalised ratio (INR) were determined using a semi-automated analyser and INR values were calculated using international sensitivity indices (ISI). The sensitivity, specificity, positive predictive values (PPV), negative predictive values (NPV), and likelihood ratios of the 20WBCT compared to an INR ≥ 1.4 were calculated, alongside 95% confidence intervals.

**Results:**

We enrolled 121 patients into our study, with a median age of 26 (18.0–35.0) years and a male predominance (75.2%). The 20WBCT was positive (abnormal) in 101 out of 121 patients at timepoint 0h, of which 95 had an INR ≥ 1.4, giving a sensitivity of 87.2% (95%CI 79.4–92.8). Among patients with a negative 20WBCT (normal), six had an INR < 1.4 giving a specificity of 50% (95%CI 21.1–78.9). The positive and negative likelihood ratios were 1.7 (95%CI 1.6–1.9) and 0.3 (95%CI 0.1–0.4) respectively.

**Conclusion:**

The 20WBCT is a simple, cheap, and easily accessible bedside test with a high sensitivity for the detection of patients with venom induced consumptive coagulopathy (VICC) following envenoming by *E*. *ocellatus*, although false positives do occur. Repeated 20WBCTs can identify patients with new, persistent, and rebound coagulopathy.

## Author summary

Bites by the West African carpet viper (*Echis ocellatus*) are very common in the savannah regions of west Africa and envenomed victims usually develop a bleeding syndrome. Routine laboratory coagulation tests are unavailable in health facilities where most snakebite victims are treated. We conducted analysis of 20-minute whole blood clotting tests (20WBCT) performed on snakebite patients in Kaltungo General Hospital, Nigeria who presented within 24 hours of the bite and who brought a dead snake along for snake identification, and we assessed its ability to detect venom induced consumptive coagulopathy (VICC) (defined as an INR ≥1.4). In our cohort of 121 patients, the 20WBCT had a sensitivity of 87% and a specificity of 50% in detecting VICC. Performing the 20WBCT increased the probability of detecting VICC among our study participants, and it was useful in identifying patients with persistent and rebound coagulopathies who may require extra doses of antivenom. Our findings show that the 20WBCT is a simple, cheap, and easily accessible bedside test with a high sensitivity for the detection of patients with VICC following carpet viper envenoming in North-eastern Nigeria.

## 1.0. Introduction

Snakebite envenoming is a major cause of death and disability in tropical parts of the world and is listed as a priority neglected tropical disease (NTD) by the World Health Organization (WHO) [1]. Snake species of the genus *Echis* are widely distributed in the Northern half of Africa, the Middle East, and the Indian subcontinent and are responsible for a significant proportion of snakebite deaths [2]. *Echis ocellatus* (West African carpet viper) is particularly common in the savannah region of West Africa where high rates of subsistence agriculture exposes farmers and agricultural workers to carpet viper bites especially around the farming season [3–8]. Another *Echis sp*, *Echis romani* was the predominant *Echis sp* (10% of all snakes) in a recent field survey in Chad and could probably be present in parts of West and Central Africa [9]. *Echis* venom is similar across species and contains a metalloproteinase-based prothrombin activator (ecarin) that activates prothrombin, with positive and negative feedback loops that culminate in the depletion of Factor V, Factor VIII, and fibrinogen, resulting in venom-induced consumptive coagulopathy (VICC) [10–12]. The venom also contains other coagulotoxins that can act directly on fibrinogen, hemorrhagins that can cause direct damage to the blood vessel wall, and other toxins that disrupt the integrity of the plasma membranes of muscle fibres, contributing to tissue damage [10,12–14]. Envenoming causes local pain and swelling but is dominated by a coagulopathy and bleeding syndrome that can involve spontaneous systemic bleeding from the gums, gastrointestinal, and genitourinary tracts, recent trauma sites or old wounds. Some patients may present with subarachnoid or cerebral haemorrhages in severe cases [4,7,13]. Early diagnosis of envenoming is crucial in the management of such snakebite victims as it allows for early antivenom administration, neutralisation of venom toxins and resynthesis of consumed coagulation factors.

Laboratory coagulation tests such as prothrombin time (PT)/ international normalised ratio (INR) and the fibrinogen concentration can detect coagulation abnormalities following envenoming by some snake species [8]. They have been shown to be the most effective way of diagnosing and monitoring VICC in snakebite patients and in determining the need for antivenom treatment [15]. However, these tests are not widely available in rural hospitals in sub-Saharan Africa (SSA) or other lower resource settings where snakebite is most common.

Bedside clotting tests have been used since 1913 to diagnose haematological abnormalities and to monitor anticoagulant treatment, often being described as simple and easy bedside tests [16]. The 20-minute whole blood clotting test (20WBCT), was introduced in 1977 by Warrell and colleagues, as an easy and sensitive sign to detect life threatening haemotoxic envenoming [7]. The test was originally described as placing “a few millilitres of freshly sampled venous blood” into a new, clean, dry glass vessel (tube or bottle), and leaving it undisturbed for 20 minutes at ambient temperature. The vessel is tipped once at 20 minutes, and if the blood has not clotted and runs out, this indicates the presence of VICC and a positive test [7]. The WHO, alongside Ministries of Health of several countries, currently recommend the 20WBCT as a bedside test to detect clotting abnormalities associated with snakebite envenoming and to determine the need for antivenom therapy [17,18]. Several studies have tried to validate the 20WBCT for detecting snakebite envenoming caused by various snake species found in several countries outside SSA. Compared to formal coagulation tests such as the INR and fibrinogen concentration, the sensitivity of the 20WBCT varied between 31–100%, and the specificity between 89–100%, largely due to heterogeneity in study setting and design [19–23]. When compared to an INR > 1.4, a standardised 20WBCT had a sensitivity of 82% with a specificity of 98% among patients envenomed by Russell’s vipers (*Daboia russelii*) in Sri Lanka [24]. The 20WBCT has never been validated against the INR for diagnosing VICC following bites by *E*. *ocellatus*. In Kaltungo General Hospital in Nigeria, over 90% of snakebite patients present with clinical features compatible with envenoming by *E*. *ocellatus*, and about 40% of the victims bring along the dead snake for identification [25,26].

The aim of this study was to investigate the diagnostic characteristics of the 20WBCT in identifying VICC and informing the use of antivenom following snakebite envenoming by *E*. *ocellatus*. Specifically, the study sought to assess the sensitivity, specificity, positive predictive value, negative predictive value and likelihood ratios of the 20WBCT compared to an INR ≥1.4 following envenoming by *E*. *ocellatus*. We also explored the ability of the 20WBCT to detect incomplete restoration of clotting ability after antivenom treatment.

## 2.0. Methods

### 2.1. Ethics statement

The study was approved by competent authorities at the Gombe State Ministry of Health (reference number MOH/ADM/621/VOL.I/143) and the Research Ethics Committee of the Liverpool School of Tropical Medicine (reference number 19–063). Written informed consent was obtained from the participants prior to data collection, and a witnessed verbal consent was obtained for participants who could not read or write. For participants less than 18 years of age, their assent was sought directly, and written or witnessed verbal consent were obtained from their legal guardians.

### 2.2. Design and setting

#### 2.2.1. Study setting and study population

This prospective cohort study was conducted at Kaltungo General Hospital (KGH), Gombe State, Nigeria. Located in the savannah region of North-Eastern Nigeria, the hospital is a specialised centre with expertise in snakebite research and training [25,26].

Patients were enrolled between September 2019 and September 2021. We included patients who presented within 24 hours of the snakebite, were aged ≥ 10 years, brought a dead carpet viper and provided consent/assent to participate in the study. The carpet vipers in this study were identified as *Echis ocellatus*, but recent herpetological surveys suggest they could likely be *Echis romani* [9,27]. We have nonetheless maintained the carpet viper specie as *Echis ocellatus* throughout the study while awaiting further evidence of the reclassification of snakes from around KGH.

#### 2.2.2. Current practice at KGH

Current practice involves the use of the 20WBCT on arrival to identify patients with coagulopathy–which is diagnostic of carpet viper envenoming in this setting–and to guide clinical management and antivenom administration. The test is usually repeated every 6 hours following antivenom administration until clotting is restored, and then every 24 hours for at least three days. There are no formal guidelines on the diagnosis or management of snakebite envenoming in KGH, however, the staff have benefited from years of exposure to snakebite clinical research, including randomised controlled clinical trials, and they have adapted best practices from WHO guidelines and other relevant publications into the way they manage snakebite victims [17,25].

All patients were treated with EchiTAb-Plus-ICP antivenom (a trivalent anti *Echis ocellatus*, *Bitis arietans* and *Naja nigricollis* specific, whole equine immunoglobulin (IgG), antivenom manufactured by Instituto Clodomiro Picado, Costa Rica) according to standard of care, irrespective of their participation in the study. Disruptions in antivenom supply during the study caused the attending physicians to reduce the doses of antivenom administered, and there was a complete antivenom stockout for about 6 months. Patient enrolment was suspended during this period because exposing patients to multiple blood draws in the absence of antivenom treatment was deemed unethical by the study team.

### 2.3. Data collection and procedures

#### 2.3.1. Data collection

Data were collected on demographics, bite circumstances, bite characteristics, important time variables (time of bite, time of hospital arrival, time of sample collection), 20WBCT results, and antivenom treatment (other clinical indications, administration times, brand, dosage, adverse reactions). Blood samples were collected at the following timepoints: 0 hour (admission), 6 hours, and then every 24 hours up until discharge or the tenth day of admission.

#### 2.3.2. 20WBCT procedure

Two millilitres of fresh whole blood were placed in a 5 ml clean, new, dry, plain glass tube and left undisturbed for 20 minutes at room temperature. The tube was then tipped to see if the blood ran out or had clotted. The test was reported positive (abnormal) if the blood ran out after 20 minutes and negative (normal) if the blood failed to run out [17]. The test was conducted by doctors and nurses working at KGH. They routinely conduct the 20WBCT on snakebite victims and they received training on standardised 20WBCT procedures, delivered by two authors (MH and AH) with significant clinical and research experience on snakebite envenoming.

#### 2.3.3. Prothrombin time and INR

Blood samples were collected in 3.2% buffered sodium citrate vacutainer tubes (BD Vacutainer) maintaining a whole-blood-to-anticoagulant ratio of 9:1. Prothrombin time (PT) was determined using the semi-automated blood coagulation analyzer Start Max (STAGO, UK) according to the manufacturer’s specifications. The INR values were calculated using the international sensitivity indices (ISI) for each lot of PT reagents [28]. The 20WBCT and INR per time point were based on the blood collected in one venepuncture using different tubes for each test.

### 2.4. Sample size

A previous study comparing the 20WBCT to an INR > 1.4 found a sensitivity of 82% for patients presenting with Russell’s viper bites in Sri Lanka [24]. We estimated a minimum sample size of 177 participants to detect a sensitivity of 90% of the 20WBCT compared to an INR ≥1.4 in detecting VICC in patients presenting with carpet viper bites, with 90% power and a type I error rate of 0.05.

### 2.5. Statistical analysis

Descriptive analysis of sociodemographic, clinical data and laboratory data was carried out using proportions, means, and standard errors (SEs) for normally distributed variables and medians and interquartile ranges (IQRs) for nonparametric variables. The results of the 20WBCT and the INR were compared at timepoint 0hr, and a Fisher’s exact test was used to test statistical significance. The sensitivity, specificity, positive predictive values (PPV), negative predictive values (NPV), and likelihood ratios of the 20WBCT in detecting VICC compared to an INR ≥ 1.4 were calculated, alongside 95% confidence intervals. Pre- and post-test probabilities were calculated for a positive 20WBCT (abnormal). Sensitivity analyses were carried out using different cut-offs for INR (1.2 and 1.6). Stata V16.1 was used for statistical analyses and a p-value <0.05 was used for statistical significance. The Standards for Reporting of Diagnostic Accuracy Studies (STARD) guidelines were used to report our findings, and a STARD checklist and flow chart have been included as a supplementary material (S1 Table and S1 Fig).

## 3.0. Results

### 3.1. Sociodemographic characteristics

During the study period, 5358 patients presented to KGH with a snakebite. A detailed breakdown of the reasons for the exclusion of 5237 patients was unavailable, but anecdotal reports from the attending clinicians suggest that failure to bring the dead carpet viper and presenting later than 24 hours after the bite were the main reasons for exclusion. Consequently 121 patients were enrolled into our study (S1 Fig). The median age was 26 (18.0–35.0) years, most patients were males (75.2%), and farming was the predominant occupation (44.6%). Table 1 presents the sociodemographic and bite characteristics of the study participants.

**Table 1:** Sociodemographic and bite characteristics of study participants.

Characteristics (N = 121)	Frequency	Percent (%)
**Age** Age, median (IQR)	26 (18–35)	
**Gender**		
Male	91	75.2
Female	30	24.8
**Formal education** *		
None	55	45.5
Primary	21	17.4
Secondary	41	33.9
Tertiary	3	2.5
**Marital status**		
Single	47	38.8
Married	74	61.2
**Occupation** *		
Farmer	54	44.6
Herder	15	12.4
Business/Artisan/Public servant	16	13.2
Students/Unemployed	33	26.3
**Previous snakebite**	18	14.9
**Bite site** *		
Lower back	3	2.5
Lower limb	98	80.9
Upper limb	19	15.6

*Total number of participants is less than 121. Formal education& Bite site– 1(0.8%) missing. Occupation 3(2.5%) missing

### 3.2. Time to hospital presentation

The median delay from bite to hospital arrival was 4.5 (2.8–8.9) hours. Patients whose 20WBCT was negative (normal) on arrival had a shorter median delay of 3.1 (2.1–3.8) hours, compared to a delay of 5.3 (3.0–10.1) hours for patients with a positive 20WBCT (abnormal).

### 3.3. Diagnostic test characteristics of the 20WBCT at time point 0 hour

The 20WBCT was positive (abnormal) in 101 out of 121 patients at timepoint 0h, of which 95 had an INR ≥ 1.4, giving a sensitivity of 87.2% (95%CI: 79.4–92.8) and a specificity of 50.0% (95%CI: 21.1–78.9) (See Table 2). This sample of patients had a pre-test probability of 90%, and the positive predictive value of a positive 20WBCT (abnormal) on admission was 94.1% (95%CI 87.5–97.8). After performing the 20WBCT, the probability of having VICC (post-test probability) increased from 90% to 94%.

**Table 2:** Sensitivity, Specificity, Positive predictive and Negative predictive values of 20WBCT vs INR ≥ 1.4, n = 121.

20WBCT at time 0hr	INR		
	INR ≥ 1.4	INR < 1.4	Total
Positive (abnormal)	95	6	101
Negative (normal)	14	6	20
Total	109	12	121
Sensitivity	87.2% (95%CI: 79.4–92.8)		
Specificity	50.0% (95%CI: 21.1–78.9)		
Positive predictive value	94.1% (95%CI: 87.5–97.8)		
Negative predictive value	30.0% (95%CI: 11.9–54.3)		
Likelihood ratio for +ve test	1.7 (95%CI: 1.6–1.9)		
Likelihood ratio for -ve test	0.3 (95CI%: 0.1–0.4)		

### 3.4. Diagnostic use of the 20WBCT at other time points

#### 3.4.1. Newly positive 20WBCT (abnormal)

Eleven of the 20 (55%) patients with a negative 20WBCT (normal) on admission subsequently developed a positive 20WBCT (abnormal) at 6h. Two (10%) patients and one (5%) patient further developed positive (abnormal) tests at 24h, and 48h respectively. There were no new positive 20WBCTs (abnormal) beyond 48h post admission. Only two patients had a consistently negative 20WBCT (normal) throughout admission. These two patients had INRs ranging from 0.81 to 6.5, with one patient having an INR ≥1.4 on three separate occasions (4.68, 2.99 and 1.48), and the other patient having an INR ≥1.4 on two separate occasions (6.5 and 1.49).

#### 3.4.2. Persistently positive 20WBCT (abnormal)

The 20WBCT was persistently positive (abnormal) in 48 (40.4%) patients at 6h and in 11 (9.4%), six (5.3%) and eight (6.9%) at 24h, 48h and 72h respectively, despite antivenom treatment. The median INRs for patients with persistently positive 20WBCT (abnormal) at time points 6h and 48 h were 4.8 and 5.2 respectively and seven (11.8%) patients had an INR < 1.4 and a positive 20WBCT (abnormal). At time points 72h and 96h, the median INRs were 1.3 and 1.1 respectively, and 10 (58.8%) patients had an INR < 1.4 and a positive 20WBCT (abnormal).

#### 3.4.3. Rebound positive 20WBCT (abnormal)

The 20WBCT detected rebound coagulopathy at 24h with two (1.7%) patients having a coagulopathy (positive 20WBCT (abnormal)) after an initial favourable response to antivenom treatment. A rebound positive 20WBCT (abnormal) was also detected in eight (7.1%), eight (6.9%), and six (5.6%) patients at 48h, 72h and 96h respectively. For patients with rebound positive 20WBCT (abnormal), the median INRs at timepoints 24h, 48h, and 96h were 5.1, 3.8 and 3.7 respectively, and seven (29.2%) patients had an INR< 1.4 and a positive 20WBCT (abnormal) (See Table 3).

**Table 3:** 20WBCT results and patients with new, persistent, and rebound positive 20WBCT (abnormal) at various time points.

Time points	0h	6h	24h	48h	72h	96h
20WBCT						
Negative (normal)	20(16.5%)	60(50.4%)	102(87.2%)	98(86.7%)	99(86.1%)	93(86.1%)
Positive (abnormal)	101(83.5%)	59(49.6%)	15(12.8%)	15(13.3%)	16(13.9)	15(13.9)
Newly positive	101(83.5%)	11(9.2%)	2(1.7%)	1(0.9%)	0	0
Persistent positive*	0	48(40.4%)	11(9.4%)	6(5.3%)	8(6.9%)	9(8.3%)
INR, median (iqr)	-	4.8(2.7–6.5)	1.7(1.1–6.5)	5.2(1.3–6.5)	1.3(1.1–6.5)	1.1(1.0–6.5)
Rebound positive^	0	0	2(1.7%)	8(7.1%)	8(6.9%)	6(5.6%)
INR, median (iqr)	-	-	5.1(3.8–6.5)	3.8(1.6–6.5)	2.4(1.5–5.2)	3.7(1.2–6.5)
Total		121	119	117	113	115	108

* = A positive 20WBCT (abnormal) at the previous timepoint immediately preceding the current positive 20WBCT

^ = A previous positive 20WBCT (abnormal) at any time point, followed by a negative 20WBCT (normal) immediately preceding the current positive 20WBCT

### 3.5. Antivenom treatment

One hundred and sixteen patients (95.9%) received EchiTAb-Plus-ICP antivenom in this study. The indications for antivenom were reported as a positive 20WBCT (abnormal) in 109 (93.9) patients, spontaneous bleeding in four (3.5%) patients and rapidly progressive swelling in three (2.6%) patients. In 60 (52.17%) patients, there was more than one indication for antivenom, the most common being a positive 20WBCT (abnormal) and rapidly progressive swelling in 54 (46.9%) patients. Treatment doses were reduced from the recommended three vials of EchiTAb-Plus-ICP per dose, to one vial in 94 (81.0%) patients and two vials in 16 (13.8%) patients. Only five (4.3%) patients received the recommended three vials of antivenom, and one patient received four vials of antivenom in a single dose. Eighteen (15.5%) patients received a second dose of antivenom. The median time between the first and second doses was 39.8h (IQR, 25.1–62.6h). Seven (38.9%) of these 18 patients had a persistently positive 20WBCT (abnormal) and eight (44.4%) had a rebound positive 20WBCT (abnormal). Four patients (3.5%) received three doses of antivenom, two had a persistently positive 20WBCT (abnormal) and two had a rebound positive 20WBCT (abnormal). One patient received four doses of antivenom, and the patient had a persistently positive 20WBCT (abnormal) up to 96h post admission. There was no stand-alone clinical indication for a repeat antivenom dose. Median time from bite to antivenom administration was 6.4h (IQR 3.9–11.8h) and 17 (14.6%) patients had their antivenom delayed due to disruptions in availability of antivenom. Twelve (10.4%) patients developed acute adverse reactions to antivenom treatment, with five (4.3%) cases reported as facial oedema, three (2.6%) cases as urticaria, two (1.7%) cases as cyanosis and one (0.9%) case each as abdominal pain and vomiting. Of the five patients who did not receive antivenom during this admission, all had a negative 20WBCT (normal) on arrival, with two maintaining a negative 20WBCT (normal) throughout admission.

### 3.6. Sensitivity analysis

The sensitivity and specificity of the 20WBCT were 86.6% (95%CI: 78.9–92.3) and 55.6% (95%CI: 21.2–86.3) respectively when using an INR cut-off of 1.2 to detect VICC, and 86.9% (95%CI: 79.0–92.7) and 42.9% (95%CI: 17.7–71.1) respectively for an INR cut-off of 1.6 (see S2 and S3 Tables). The prevalence of VICC (pre-test probabilities) using INR cut-offs of 1.2 and 1.6 were 93% and 88% respectively, and after performing the 20WBCT, the probabilities of having VICC (post-test probabilities) increased to 96% and 92% respectively.

## 4.0. Discussion

In Kaltungo General Hospital, Gombe State, Nigeria, patients who presented with a snakebite and brought along the dead carpet viper had a 90% probability of developing VICC (defined as INR ≥ 1.4). The 20WBCT had a sensitivity of 87% and a specificity of 50% in detecting VICC, and the test increased the probability of diagnosing VICC by four percentage points. The 20WBCT was also useful in the detection of new onset coagulopathy post admission, persistent coagulopathy and rebound coagulopathy.

Our sensitivity of 87% is higher than findings by Ratnayake et al, Silva et al, and Dsilva et al, who found sensitivities of 82%, 54% and 73% respectively when comparing the 20WBCT to INR > 1.4 [19,24,29,30], but lower than findings by Sharma et al, Bhatt et al and Shenoy, with 89%, 95% and 96% respectively [30–32]. A head-to-head comparison of the sensitivities of these studies does not provide an accurate assessment of the performance of the test due to heterogeneity in study setting, snake species, INR cut-offs for defining VICC, study inclusion criteria, sample collection time points and other design and analytical methods. It is not clear to what extent the different mechanisms of VICC by different species affect the performance of the 20WBCT.

*E*. *ocellatus* venom contains prothrombin activators which convert prothrombin to meizothrombin and which have positive and negative feedback loops which end up with deficiencies in Factors II, V, VIII and fibrinogen [10]. Meizothrombin is a potent activator of Factor V, rapidly depleting Factor V levels and causing a prolongation of PT and a high INR [33]. For snakebite envenoming, an assumption is made that all patients (not on anticoagulants) have a normal clotting profile and that a minor deviation from the normal is indicative of a rising INR following envenoming, and if left untreated, could result in significant coagulopathy. Nonetheless, there remains a need for a more informed choice of the INR cut-off to indicate VICC following envenoming, a better understanding of the change in INR after antivenom treatment, and for identifying potential endpoints/cut-offs to indicate reversal of coagulopathy or resolution of VICC.

Bedside clotting tests such as the 20WBCT correlate closely with the fibrinogen concentration and are more likely to be positive (abnormal) with partial or complete fibrinogen depletion, which are endpoints of VICC [34,35]. This makes the duration of the test a crucial factor in its interpretation. The scientific justification for the use of 20 minutes in the 20WBCT is unclear; several studies have experimented with different observation times for the WBCT, ranging between 10 and 30 minutes with no justification for the different choices [4,36,37].

In our study, 13.1% (n = 14) of the patients with VICC had a false negative 20WBCT (negative 20WBCT (normal) with an INR ≥ 1.4) on admission and would not have received antivenom had the decision been based solely on the 20WBCT. Of these patients, 7 (50%) had a clinical indication for antivenom, despite the negative 20WBCT (normal), and received antivenom within 6 hours of admission. Other studies comparing 20WBCT to INR report false negative rates ranging from 4% to 46.2% [19,29–32]. This suggests that, if used in isolation, the 20WBCT may miss a substantial number of patients who may have mild/moderate envenoming, or who may be early in the envenoming process. Fifty percent (n = 6) of the patients without VICC had a false positive 20WBCT (positive 20WBCT (abnormal) with an INR < 1.4) on admission. All of these patients eventually had an INR ≥ 1.4 at later timepoints.

Our specificity of 50% for the 20WBCT is the lowest that has been reported in the recent literature. Our sample had a very high prevalence of VICC (90%) and few true negatives on admission, giving a wide confidence interval around the 50% estimate, and this methodological design limited our ability to reliably assess the specificity of the 20WBCT. The true specificity requires evaluation of a population bitten by a wider variety of species, including species which do not cause coagulopathy. Other studies report very high specificities of the 20WBCT compared to INR > 1.4, ranging from 94–100% [24,29,30].

We observed false negative 20WBCT and false positive 20WBCT on admission, with all false negative 20WBCT subsequently becoming positive (abnormal) and all false positive 20WBCT recording high INRs at later time points. This suggests that the time since the bite may influence the results of the 20WBCT, particularly for false negative 20WBCT, with patients who present early after the bite being at risk of having their coagulopathies missed. A false negative 20WBCT could delay antivenom therapy and expose patients to further complications of envenoming. A false positive diagnosis may expose patients to adverse reactions of antivenoms, increase health expenditure and use up an often-limited stock of antivenom [30]. These findings also highlight the complexity of snakebite envenoming. It is a rapid and dynamic process, which can be influenced by factors which are unknown at the time of admission, such as; the amount of venom injected, venom distribution at time of admission (venom depots) and the rate of clotting factor replenishment following neutralisation of venom toxin [10]. These factors, in addition to variable delays in hospital presentation after a bite, require all snakebite victims to be continuously monitored following admission. This study showed that the 20WBCT is potentially valuable in monitoring the dynamic evolution of envenoming and identifying patients requiring new or additional antivenom beyond time point 0h. Repeating the 20WBCT appeared to identify new cases of VICC and to identify patients with persistent and rebound coagulopathies. The PT/INR are more effective at identifying new and persistent coagulopathies, but their significance in clinical decision making, especially for persistent and rebound coagulopathies, requires further research on the relationship between change in INR over time, risk of bleeding, and the need for additional doses of antivenoms [15,30]. Previous studies have shown that the 20WBCT can reliably indicate severe hypofibrinogenemia and afibrinogenemia, thereby estimating the risk of bleeding [34,35], however, several aspects of the 20WBCT require further research, scientific justifications, and standardization, such as the size of the test tube, the volume of blood, the effect of different room temperatures, the duration of the test and the effect of the time since the bite. Current clinical practice at KGH relies mainly on the 20WBCT for indicating additional antivenom therapy, and this study supports this practice based on the 20WBCT’s ability to detect persistent and rebound coagulopathy.

In addition to its clinical prowess in detecting VICC, compared to the INR, the 20WBCT is inexpensive as it can be conducted using any clean glass vial. Health facilities in resource-constrained areas can preserve glass vials to conduct 20WBCT, enabling patients save up for antivenom and other treatment costs. Furthermore, in some laboratories, clotting assays are run in batches, with delays of up to 12 hours between sample collection and results. The 20WBCT is timesaving, can be conducted at any point and can be used as an extra element for the close monitoring required for all snakebite patients, especially those who have been administered antivenom.

The 20WBCT remains a simple and practical way of identifying the majority of patients with VICC on admission following E. ocellatus bites and supporting clinical decision making for additional antivenom treatment in resource-constrained settings, albeit with some inconsistencies and shortcomings. Future research, focussing on clinical outcomes and point-of-care coagulopathy assays could increase diagnostic options for VICC and improve clinical decision making. Rapid diagnostic tests, with their simple design and short reading times, can be a useful addition to current diagnostic options for VICC [38,39]. The ideal test would; combine the affordability and practicality of the 20WBCT with the accuracy of the PT/INR, detect envenoming early, and reliably assess recovery following antivenom therapy. The minimum observation period following a suspected/confirmed carpet viper bite may require reconsideration given that one of our participants developed a new positive 20WBCT (abnormal) 48 hours after admission.

### Strengths and limitations

To the best of our knowledge, this is the first study to formally assess the diagnostic characteristics of the 20WBCT (vs INR) for carpet viper envenoming. However, our results should be interpreted with the following limitations in mind. We were unable to meet our minimum sample size and consequently, our study was underpowered, in part due to disruptions in antivenom supply. Our study design selected for patients with a high probability of being envenomed by carpet vipers, as such, we did not have enough true negatives in our study population, and this limited our ability to assess the specificity of the 20WBCT or to assess its value in diagnosing systemic envenoming in general clinical practice. Furthermore, only 2% of snakebite victims were eligible for inclusion in our study, with consequences on the external validity of our study. Nonetheless, our study provides new insight into the characteristics of the 20WBCT in detecting carpet viper envenoming in north-eastern Nigeria.

## 5.0. Conclusion

The 20WBCT is a simple, cheap, and easily accessible bedside test with a high sensitivity for the detection of patients with VICC following envenoming by *E*. *ocellatus* (West African carpet viper), although false positives do occur. Repeated 20WBCT can identify patients with new, persistent, and rebound coagulopathy, thereby extending the utility of the 20WBCT to monitoring recovery of normal coagulation and identifying the need for further antivenom treatment.

## Supporting information

S1 Table Standards for Reporting of Diagnostic Accuracy Studies Checklist. (DOCX)

S2 Table Sensitivity, Specificity, Positive predictive and Negative predictive values of 20WBCT vs INR ≥ 1.2. (DOCX)

S3 Table Sensitivity, Specificity, Positive predictive and Negative predictive values of 20WBCT vs INR ≥ 1.6. (DOCX)

S1 Fig Standards for Reporting of Diagnostic Accuracy Studies patient flow chart. (DOCX)

## References

[1] World Health Organisation. Snakebite envenoming: a strategy for prevention and control. (2019). Available from: https://www.who.int/publications/i/item/9789241515641 (Assessed 12/10/2022).

[2] Warrell DA. Clinical toxicology of snake bite in Africa and the Middle East/Arabian Peninsula. In Handbook of: Clinical Toxicology of Animal Venoms and Poisons 433–492 (CRC Press, 2017).

[3] Chippaux J-P, Kambewasso A. Snake bites and antivenom availability in the urban community of Niamey, Niger. *Bulletin de la Societe de Pathologie Exotique (1990)* 95, 181–183 (2002). 12404866

[4] Chippaux J-P, Rage-Andrieux V, Charrondière M, Sagot P, Lang J. Epidemiology of snake envenomations in northern Cameroon. *Bulletin de la Societe de Pathologie Exotique (1990)* 95, 184–187 (2002). 12404867

[5] Fourn L, Adè G, Fayomi E, Zohoun T. Epidemiological aspects of snakebites in Benin. *Bulletin de la Societe de Pathologie Exotique (1990)* 98, 291–292 (2005). 16402578

[6] Guyavarch E, Trape J. The incidence of snakebite in a rural zone of southeastern Senegal. *Bulletin de la Societe de Pathologie Exotique (1990)* 98, 197–200 (2005). 16267960

[7] Warrell DA, Davidson NMcD, Greenwood BM, Ormerod LD, Pope HM, Watkins BJ et al. Poisoning by bites of the saw-scaled or carpet viper (Echis carinatus) in Nigeria. *The Quarterly journal of medicine* 46, 33–62 (1977). 866568

[8] Warrell DA, Pope HM, Prentice CRM. Disseminated Intravascular Coagulation Caused by the Carpet Viper (Echis carinatus): Trial of Heparin. *British Journal of Haematology* 33, 335–342, (1976). doi: 10.1111/j.1365-2141.1976.tb03549.x 1276079

[9] Trape J-F, Kodindo ID, Djiddi AS, Mad-ToïnguéJ, Kerah CH. The snakes of Chad: results of a field survey and annotated country-wide checklist. *Bonn zoological Bulletin* 69, 367–393 (2020).

[10] Berling I, Isbister GK. Hematologic Effects and Complications of Snake Envenoming. *Transfusion Medicine Reviews* 29, 82–89, (2015). doi: 10.1016/j.tmrv.2014.09.005 25556574

[11] Isbister GK, Scorgie FE, O’Leary MA, SELDON M, Brown SGA, Lincz LF et al. Factor deficiencies in venom-induced consumption coagulopathy resulting from Australian elapid envenomation: Australian Snakebite Project (ASP-10). *Journal of Thrombosis and Haemostasis* 8, 2504–2513, (2010). doi: 10.1111/j.1538-7836.2010.04050.x 20831619

[12] McCleary RJR, Kini RM. Snake bites and hemostasis/thrombosis. *Thrombosis Research* 132, 642–646, (2013). doi: 10.1016/j.thromres.2013.09.031 24125598

[13] Gutiérrez JM, Calvete JJ, Habib AG, Harrison RA, Williams DJ, Warrell DA. Snakebite envenoming. *Nature Reviews Disease Primers* 3, 17063, doi: 10.1038/nrdp.2017.63 (2017). 28980622

[14] Ainsworth S, Slagboom J, Alomran N, Pla D, Alhamdi Y, King SI et al. The paraspecific neutralisation of snake venom induced coagulopathy by antivenoms. *Communications Biology* 1, 34, doi: 10.1038/s42003-018-0039-1 (2018). 30271920 PMC6123674

[15] Wedasingha S, Isbister G, Silva A. Bedside Coagulation Tests in Diagnosing Venom-Induced Consumption Coagulopathy in Snakebite. *Toxins* 12, 583, doi: 10.3390/toxins12090583 (2020). 32927702 PMC7551701

[16] Lee RI, White PDA. Clinical Study of the Coagulation Time of Blood. *The American Journal of the Medical Sciences (1827–1924)* 145, 495 (1913).

[17] World Health Organisation 2010. Guideline for the prevention and clincial management of snakebite in Africa. (WHO Regional Office for Africa, Mauritius, 2010).

[18] Kenya Ministry of Health. Guidelines for prevention, Diagnosis and Management of Snakebite Envenoming in Kenya (Kenya Ministry of Health, Nairobi, 2019).

[19] Dsilva AA, Basheer A, Thomas K. Snake envenomation: is the 20 min whole blood clotting test (WBCT20) the optimum test for management? *QJM*: *An International Journal of Medicine* 112, 575–579, doi: 10.1093/qjmed/hcz077 (2019). 30918965

[20] Isbister GK, Currie BJ. Suspected snakebite: One year prospective study of emergency department presentations. *Emergency Medicine* 15, 160–169, (2003). doi: 10.1046/j.1442-2026.2003.00434.x 12675626

[21] Thongtonyong N, Chinthammitr Y. Sensitivity and specificity of 20-minute whole blood clotting test, prothrombin time, activated partial thromboplastin time tests in diagnosis of defibrination following Malayan pit viper envenoming. *Toxicon*: *official journal of the International Society on Toxinology* 185, 188–192, (2020). doi: 10.1016/j.toxicon.2020.07.020 32712023

[22] Tongpoo A, Niparuck P, Sriapha C, Wananukul W, Trakulsrichai S. Utility of Thrombin Time in Management of Patients with Green Pit Vipers Bite. *SAGE Open Med* 8, 2050312120966468–2050312120966468, doi: 10.1177/2050312120966468 (2020). 35154756 PMC8826260

[23] Wijewickrama ES, Gooneratne LV, Gnanathasan A, Gawarammana I, Gunatilake M, Isbister GK. Severe acute kidney injury following Sri Lankan Hypnale spp. envenoming is associated with thrombotic microangiopathy. *Clinical Toxicology* 59, 296–302, doi: 10.1080/15563650.2020.1810695 (2021). 32870056

[24] Ratnayake I, Shihana F, Dissanayake DM, Buckley NA, Maduwage K, Isbister GK. Performance of the 20-minute whole blood clotting test in detecting venom induced consumption coagulopathy from Russell’s viper (Daboia russelii) bites. *Thromb Haemost* 117, 500–507 (2017). doi: 10.1160/TH16-10-0769 28150853

[25] Abubakar IS, Abubakar SB, Habib AG, Nasidi A, Durfa N, Yusuf PO et al. Randomised Controlled Double-Blind Non-Inferiority Trial of Two Antivenoms for Saw-Scaled or Carpet Viper (Echis ocellatus) Envenoming in Nigeria. *PLOS Neglected Tropical Diseases* 4, e767, doi: 10.1371/journal.pntd.0000767 (2010). 20668549 PMC2910709

[26] Habib A. G. & Abubakar S. B. Factors affecting snakebite mortality in north-eastern Nigeria. *International Health* 3, 50–55, doi: 10.1016/j.inhe.2010.08.001 (2011). 24038050

[27] Uetz P, Freed P, Aguilar R, Hošek J. The Reptile Database. http://www.reptile-database.org. (2022).

[28] Manning R, Laffan MA. Investigation of haemostasis. In: Bain B J, Bates I, Laffan M A. Dacie and Lewis Practical Haematology. 12th ed. Netherlands: Elsevier; 2016.P. 465.

[29] Silva A, Sarathchandra C, Senanayake H, Weerawansa P, Siribaddana S, Isbister G. Capillary blood clotting time in detecting venom-induced consumption coagulopathy (VICC).Abstract. Clin Toxicol (Phila). 2018;56 (10):919.

[30] Lamb T, Abouyannis M, de Oliveira SS, Shenoy K R, Geevar T, Zachariah A et al. The 20-minute whole blood clotting test (20WBCT) for snakebite coagulopathy—A systematic review and meta-analysis of diagnostic test accuracy. *PLOS Neglected Tropical Diseases* 15, e0009657, doi: 10.1371/journal.pntd.0009657 (2021). 34375338 PMC8405032

[31] Bhatt N, Singh A, Sharma SK. Case Report: Management of Pit Viper Envenoming without Antivenom: A Case Series. *The American Journal of Tropical Medicine and Hygiene* 102, 1440–1442, doi: 10.4269/ajtmh.20-0035 (2020). 32228791 PMC7253110

[32] Rachana Shenoy K. *Clinical and laboratory spectrum of Venom-induced consumption coagulopathy (VICC) and bedside diagnostic tests in haemotoxic snakebite in a tertiary care centre in South India*: *ProTIV Study*, Christian Medical College, Vellore, (2019).

[33] Tans G, Nicolaes GA, Thomassen MC, Hemker HC, van Zonneveld AJ, Pannekoek H et al. Activation of human factor V by meizothrombin. *The Journal of biological chemistry* 269, 15969–15972 (1994). 8206891

[34] Benjamin JM, Chippaux J-P, Sambo BT, Massougbodji A. Delayed double reading of whole blood clotting test (WBCT) results at 20 and 30 minutes enhances diagnosis and treatment of viper envenomation. *Journal of Venomous Animals and Toxins including Tropical Diseases* 24, 14, doi: 10.1186/s40409-018-0151-1 (2018). 29796013 PMC5956810

[35] Sano-Martins IS, Fan HW, Castro SC, Tomy SC, Franca FO, Jorge MT, et al. Reliability of the simple 20 minute whole blood clotting test (WBCT20) as an indicator of low plasma fibrinogen concentration in patients envenomed by Bothrops snakes. Butantan Institute Antivenom Study Group. *Toxicon*: *official journal of the International Society on Toxinology* 32, 1045–1050, doi: 10.1016/0041-0101(94)90388-3 (1994). 7801340

[36] Premawardena AP, Seneviratne SL, Gunatilake SB de Silva HJ. Excessive fibrinolysis: the coagulopathy following Merrem’s hump-nosed viper (Hypnale hypnale) bites. *Am J Trop Med Hyg* 58, 821–823, doi: 10.4269/ajtmh.1998.58.821 (1998). 9660472

[37] Srimannarayana J, Dutta TK, Sahai A & Badrinath S. Rational use of anti-snake venom (ASV): trial of various regimens in hemotoxic snake envenomation. *The Journal of the Association of Physicians of India* 52, 788–793 (2004). 15909856

[38] Harrison RA, & Gutiérrez JM. Priority Actions and Progress to Substantially and Sustainably Reduce the Mortality, Morbidity and Socioeconomic Burden of Tropical Snakebite. *Toxins (Basel)* 8, doi: 10.3390/toxins8120351 (2016). 27886134 PMC5198546

[39] Knudsen C, Jürgensen JA, Føns S, Haack AM, Friis RUW, Dam SH, et al. Snakebite Envenoming Diagnosis and Diagnostics. *Frontiers in immunology* 12, 661457, doi: 10.3389/fimmu.2021.661457 (2021). 33995385 PMC8113877

