# Supplementary material for: Diagnostic characteristics of the 20-minute whole blood clotting test in detecting venom-induced consumptive coagulopathy following carpet viper envenoming
Source: PLoS Negl Trop Dis. 2023 Jun 26;17(6):e0011442. doi: 10.1371/journal.pntd.0011442 (PMC10328339; doi:10.1371/journal.pntd.0011442)
Supplement: S1 Table — (DOCX) [file pntd.0011442.s001.docx]

| **Supplementary Table 1 - Standards for Reporting of Diagnostic Accuracy Studies (STARD) Checklist** | | | | |
| --- | --- | --- | --- | --- |
|  | **Section & Topic** | **No** | **Item** | **Reported on page #** |
|  |  |  |  |  |
|  | **TITLE OR ABSTRACT** |  |  |  |
|  |  | **1** | Identification as a study of diagnostic accuracy using at least one measure of accuracy  (such as sensitivity, specificity, predictive values, or AUC) | 1 – Abstract includes sensitivity and specificity of 20WBCT compared to INR > 1.4 |
|  | **ABSTRACT** |  |  |  |
|  |  | **2** | Structured summary of study design, methods, results, and conclusions  (for specific guidance, see STARD for Abstracts) | 2 – Structured summary |
|  | **INTRODUCTION** |  |  |  |
|  |  | **3** | Scientific and clinical background, including the intended use and clinical role of the index test | 4 - The 20WBCT has not been validated for the diagnoses of venom induced consumptive coagulopathy for patients envenomed by Echis ocellatus sp |
|  |  | **4** | Study objectives and hypotheses | 4 - The objective of this study is to investigate the diagnostic characteristics of the 20 WBCT in identifying VICC and informing the use of antivenom following snakebite envenoming by E. ocellatus. |
|  | **METHODS** |  |  |  |
|  | *Study design* | **5** | Whether data collection was planned before the index test and reference standard  were performed (prospective study) or after (retrospective study) | 4 – This was a prospective cohort study |
|  | *Participants* | **6** | Eligibility criteria | 4 - We included patients who presented within 24 hours of the snakebite, were aged ≥ 10 years, brought a dead carpet viper and provided consent/assent to participate in the study. |
|  |  | **7** | On what basis potentially eligible participants were identified  (such as symptoms, results from previous tests, inclusion in registry) | 4 - We included patients who presented within 24 hours of the snakebite, were aged ≥ 10 years, brought a dead carpet viper and provided consent/assent to participate in the study. |
|  |  | **8** | Where and when potentially eligible participants were identified (setting, location and dates) | 4 – Patients were enrolled at the Kaltongo General Hospital between September 2019 and September 2021 |
|  |  | **9** | Whether participants formed a consecutive, random or convenience series | 4 – We included patients who met our inclusion criteria |
|  | *Test methods* | **10a** | Index test, in sufficient detail to allow replication | 5 – We described the process for conducting the 20WBCT |
|  |  | **10b** | Reference standard, in sufficient detail to allow replication | 5 – We described the methods used to obtain the INR values |
|  |  | **11** | Rationale for choosing the reference standard (if alternatives exist) | 5 – Our cut-off value was based on findings from previous studies |
|  |  | **12a** | Definition of and rationale for test positivity cut-offs or result categories  of the index test, distinguishing pre-specified from exploratory | 5 – The tube containing the patients’ blood was tipped after 20 minutes to see if the blood ran out or had clotted. The test was reported abnormal if the blood ran out after 20 minutes and normal if the blood failed to run out |
|  |  | **12b** | Definition of and rationale for test positivity cut-offs or result categories  of the reference standard, distinguishing pre-specified from exploratory | 6 – We used an INR cut-off of 1.4 to define venom induced consumptive coagulopathy |
|  |  | **13a** | Whether clinical information and reference standard results were available  to the performers/readers of the index test | 5 – The test was conducted by doctors and nurses working at KGH |
|  |  | **13b** | Whether clinical information and index test results were available  to the assessors of the reference standard | 5 – The INR was conducted in the hospital laboratory by trained technicians |
|  | *Analysis* | **14** | Methods for estimating or comparing measures of diagnostic accuracy | 6 - The sensitivity, specificity, positive predictive values (PPV), negative predictive values (NPV), and likelihood ratios of the 20WBCT in detecting VICC compared to an INR ≥ 1.4 were calculated, alongside 95% confidence intervals |
|  |  | **15** | How indeterminate index test or reference standard results were handled | 6 – We did not have indeterminate results |
|  |  | **16** | How missing data on the index test and reference standard were handled | 6 – There was no missing for the 20WBCT and INR at point 0h |
|  |  | **17** | Any analyses of variability in diagnostic accuracy, distinguishing pre-specified from exploratory | 6 - The results of the 20WBCT and the INR were compared at timepoint 0hr, and a Fisher’s exact test was used to test statistical significance. |
|  |  | **18** | Intended sample size and how it was determined | 5 - We estimated a minimum sample size of 177 participants to detect a sensitivity of 90% of the 20WBCT compared to an INR ≥1.4 in detecting VICC in patients presenting with carpet viper bites, with 90% power and a type I error rate of 0.05. |
|  | **RESULTS** |  |  |  |
|  | *Participants* | **19** | Flow of participants, using a diagram | S2 Figure 1 |
|  |  | **20** | Baseline demographic and clinical characteristics of participants | 6 – Baseline characteristics are presented in Table 1 |
|  |  | **21a** | Distribution of severity of disease in those with the target condition | 8 - The INR on admission was > 5.0 in 93 (76.9%) patients, between 1.4 – 5.0 in 16 (13.2%) patients, and < 1.4 in 12 (9.9% ) patients. |
|  |  | **21b** | Distribution of alternative diagnoses in those without the target condition | - |
|  |  | **22** | Time interval and any clinical interventions between index test and reference standard | 5 - The blood samples for both tests were collected at the same time |
|  | *Test results* | **23** | Cross tabulation of the index test results (or their distribution)  by the results of the reference standard | 8 – The results of the 20WBCT and INR are presented in Table 2 |
|  |  | **24** | Estimates of diagnostic accuracy and their precision (such as 95% confidence intervals) | 8 – The results of the 20WBCT and INR are presented in Table 2 |
|  |  | **25** | Any adverse events from performing the index test or the reference standard | - |
|  | **DISCUSSION** |  |  |  |
|  |  | **26** | Study limitations, including sources of potential bias, statistical uncertainty, and generalisability | 14 - Our study design selected for patients with a high probability of being envenomed by carpet vipers, as such, we did not have enough true negatives in our study population, and this limited our ability to assess the specificity of the 20WBCT or to assess its value in diagnosing systemic envenoming in general clinical practice. |
|  |  | **27** | Implications for practice, including the intended use and clinical role of the index test | 14 - The 20WBCT is a simple, cheap, and easily accessible bedside test with a high sensitivity for the detection of patients with VICC following envenoming by E. ocellatus (Carpet vipers), although false positives do occur. |
|  | **OTHER INFORMATION** |  |  |  |
|  |  | **28** | Registration number and name of registry |  |
|  |  | **29** | Where the full study protocol can be accessed |  |
|  |  | **30** | Sources of funding and other support; role of funders | 14 - This study was funded by the National Institute for Health Research (RH) grant entitled ‘NIHR Global Health Research Group on African Snakebite Research, Liverpool School of Tropical Medicine’ (16.137.114). The funders had no role in study design, data collection and analysis, decision to publish, or preparation of the manuscript. The views expressed are those of the author(s) and not necessarily those of the NIHR or the Department of Health and Social Care. |
|  |  |  |  |  |

**Reference**

1. Bossuyt PM, Reitsma JB, Bruns DE, Gatsonis CA, Glasziou PP, Irwig L, LijmerJG Moher D, Rennie D, de Vet HCW, Kressel HY, Rifai N, Golub RM, Altman DG, Hooft L, Korevaar DA, Cohen JF, For the STARD Group. STARD 2015: An Updated List of Essential Items for Reporting Diagnostic Accuracy Studies.
