# Supplementary material for: Diagnostic characteristics of the 20-minute whole blood clotting test in detecting venom-induced consumptive coagulopathy following carpet viper envenoming
Source: PLoS Negl Trop Dis. 2023 Jun 26;17(6):e0011442. doi: 10.1371/journal.pntd.0011442 (PMC10328339; doi:10.1371/journal.pntd.0011442)
Supplement: S2 Table — (DOCX) [file pntd.0011442.s002.docx]

Supplementary Table 2. Sensitivity, Specificity, Positive predictive and Negative predictive values of 20 WBCT vs INR ≥ 1.2, n = 121

| 20WBCT at time 0hr | INR |  |  |
| --- | --- | --- | --- |
|  | INR ≥ 1.2 | INR < 1.2 | Total |
| Positive (abnormal) | 97 | 4 | 101 |
| Negative (normal) | 15 | 5 | 20 |
| Total | 112 | 9 | 121 |
| Sensitivity | 86.6% (95%CI: 78.9 – 92.3%) | | |
| Specificity | 55.6% (95%CI: 21.2 – 86.3%) | | |
| Positive predictive value | 96.0% (95%CI: 90.2 – 98.9%) | | |
| Negative predictive value | 25.0% (95%CI: 8.7 – 49.1%) | | |
| Likelihood ratio for +ve test | 1.9 (95%CI: 1.8 – 2.1) | | |
| Likelihood ratio for -ve test | 0.2 (95CI%: 0.06 – 0.4) | | |
