# Supplementary material for: Diagnostic characteristics of the 20-minute whole blood clotting test in detecting venom-induced consumptive coagulopathy following carpet viper envenoming
Source: PLoS Negl Trop Dis. 2023 Jun 26;17(6):e0011442. doi: 10.1371/journal.pntd.0011442 (PMC10328339; doi:10.1371/journal.pntd.0011442)
Supplement: S3 Table — (DOCX) [file pntd.0011442.s003.docx]

Supplementary Table 3. Sensitivity, Specificity, Positive predictive and Negative predictive values of 20 WBCT vs INR ≥ 1.6, n = 121

| 20WBCT at time 0hr | INR |  |  |
| --- | --- | --- | --- |
|  | INR ≥ 1.6 | INR < 1.6 | Total |
| Positive (abnormal) | 93 | 8 | 101 |
| Negative (normal) | 14 | 6 | 20 |
| Total | 107 | 14 | 121 |
| Sensitivity | 86.9% (95%CI: 79.0 – 92.7%) | | |
| Specificity | 42.9% (95%CI: 17.7 – 71.1%) | | |
| Positive predictive value | 92.1% (95%CI: 84.9 – 96.5%) | | |
| Negative predictive value | 30.0% (95%CI: 11.9 – 54.3%) | | |
| Likelihood ratio for +ve test | 1.5 (95%CI: 1.4 – 1.7) | | |
| Likelihood ratio for -ve test | 0.3 (95CI%: 0.2 – 0.5) | | |
