# Supplementary material for: Diagnostic characteristics of the 20-minute whole blood clotting test in detecting venom-induced consumptive coagulopathy following carpet viper envenoming
Source: PLoS Negl Trop Dis. 2023 Jun 26;17(6):e0011442. doi: 10.1371/journal.pntd.0011442 (PMC10328339; doi:10.1371/journal.pntd.0011442)
Supplement: S1 Fig — (DOCX) [file pntd.0011442.s004.docx]

STARD diagram to report flow of participants through the study

All patients reporting to KGH with a snakebite during study period

n = 5358

Excluded/ Not enrolled in study = 5237

Presented beyond 24 hours after snakebite

Did not bring dead carpet viper

Antivenom stockout

Declined consent

INR < 1.4 at time 0h

n = 6

Enrolled participants

n = 121

20WBCT inconclusive

n = 0

Final Management

-Received antivenom = 9

-Did not receive antivenom = 5

Final Management

-Received antivenom = 101

-Did not receive antivenom = 0

20WBCT normal

n = 14

20WBCT abnormal

n = 101

Potentially eligible participants

n =

Potentially eligible participants

n =

Potentially eligible participants

n =

INR inconclusive

n = 0

INR ≥ 1.4 at time 0h

n = 95
